# Evaluation of an Ambulatory ECG Analysis Platform Using Deep Neural Networks in Routine Clinical Practice

**DOI:** 10.1161/JAHA.122.026196

**Published:** 2022-09-08

**Authors:** Laurent Fiorina, Carole Maupain, Christophe Gardella, Vladimir Manenti, Fiorella Salerno, Pierre Socie, Jia Li, Christine Henry, Audrey Plesse, Kumar Narayanan, Aurélie Bourmaud, Eloi Marijon

**Affiliations:** ^1^ Ramsay Santé Institut Cardiovasculaire Paris Sud, Hôpital privé Jacques Cartier Massy France; ^2^ AP‐HP, La Pitié Salpêtrière University Hospital, Cardiology Department Paris France; ^3^ Cardiologs® Technologies Paris France; ^4^ Université de Paris, PARCC, INSERM Paris France; ^5^ Medicover Hospitals Hyderabad India; ^6^ Assistance Publique‐Hôpitaux de Paris, Unit of Clinical Epidemiology, Robert Debré children’s hospital University of Paris Inserm U1123 and CIC‐EC Paris France; ^7^ AP‐HP, European Georges Pompidou Hospital, Cardiology Department Paris France

**Keywords:** cardiac arrhythmias, diagnosis, electrophysiology, Arrhythmias

## Abstract

**Background:**

Holter analysis requires significant clinical resources to achieve a high‐quality diagnosis. This study sought to assess whether an artificial intelligence (AI)‐based Holter analysis platform using deep neural networks is noninferior to a conventional one used in clinical routine in detecting a major rhythm abnormality.

**Methods and Results:**

A total of 1000 Holter (24‐hour) recordings were collected from 3 tertiary hospitals. Recordings were independently analyzed by cardiologists for the AI‐based platform and by electrophysiologists as part of clinical practice for the conventional platform. For each Holter, diagnostic performance was evaluated and compared through the analysis of the presence or absence of 5 predefined cardiac abnormalities: pauses, ventricular tachycardia, atrial fibrillation/flutter/tachycardia, high‐grade atrioventricular block, and high burden of premature ventricular complex (>10%). Analysis duration was monitored. The deep neural network–based platform was noninferior to the conventional one in its ability to detect a major rhythm abnormality. There were no statistically significant differences between AI‐based and classical platforms regarding the sensitivity and specificity to detect the predefined abnormalities except for atrial fibrillation and ventricular tachycardia (atrial fibrillation, 0.98 versus 0.91 and 0.98 versus 1.00; pause, 0.95 versus 1.00 and 1.00 versus 1. 00; premature ventricular contractions, 0.96 versus 0.87 and 1.00 versus 1.00; ventricular tachycardia, 0.97 versus 0.68 and 0.99 versus 1.00; atrioventricular block, 0.93 versus 0.57 and 0.99 versus 1.00). The AI‐based analysis was >25% faster than the conventional one (4.4 versus 6.0 minutes; *P*<0.001).

**Conclusions:**

These preliminary findings suggest that an AI‐based strategy for the analysis of Holter recordings is faster and at least as accurate as a conventional analysis by electrophysiologists.

ECG Holter monitoring is a common tool used for the detection and characterization of cardiac rhythm abnormalities.^1^ The conventional techniques used to analyze Holter recordings are based on signal processing and feature extraction (P wave, QRS complexes, RR intervals, etc), which allows classification of rhythms and diagnosis of abnormalities using predefined rules and feature analysis.^2^

Deep neural networks (DNNs) are an emerging diagnostic tool in cardiology.^3^ They have recently been applied to ECG analysis, having demonstrated promising results on rhythm classification accuracy in Holter ECGs.^4^, ^5^ However, the performance of DNNs for Holter analysis in routine clinical practice has not been studied yet. Before adopting a new diagnostic procedure, it is essentially important to assess whether the diagnostic accuracy of the new procedure is noninferior to that of the standard procedure.^6^ In this study, we compared the Cardiologs Holter analysis platform (version 2.1.19; Cardiologs, Paris, France), a Food and Drug Administration cleared and CE marked DNN‐based platform, with a conventional platform (Sorin Synescope, version 3.10, ElaMedical) in their ability to identify major rhythm abnormalities and in their performance indicators.

## METHODS

The data supporting the findings of this study may be made available from the corresponding author on reasonable request. This multicentric comparative study aimed principally to demonstrate the noninferiority of a DNN‐based Holter analysis solution compared with a conventional ambulatory ECG analysis platform in correctly detecting 5 major rhythm abnormalities. It secondarily aimed at comparing all performance indicators for both diagnosis procedures. This study was approved by the local ethics committee, and subjects gave informed consent.

A total of 1000 consecutive 24‐hour Holter recordings performed in adults from January to December 2018 were collected in 3 tertiary care hospitals in the Paris area. Those recordings were collected from unselected routine patients seen in the ambulatory monitoring lab of the 3 hospitals and are therefore representative, in terms of arrhythmia prevalence, of the population on which Holter recordings are usually performed. When patients had >1 recording performed, only the first one was included. Holters of patients implanted with a pacemaker or a defibrillator were excluded. All recordings were performed using a 3‐lead Holter device (Spiderview, MicroPort, Shanghai, China), with a sampling rate of 200 Hz.

### DNN‐Based Platform Workflow

**Figure 1:**
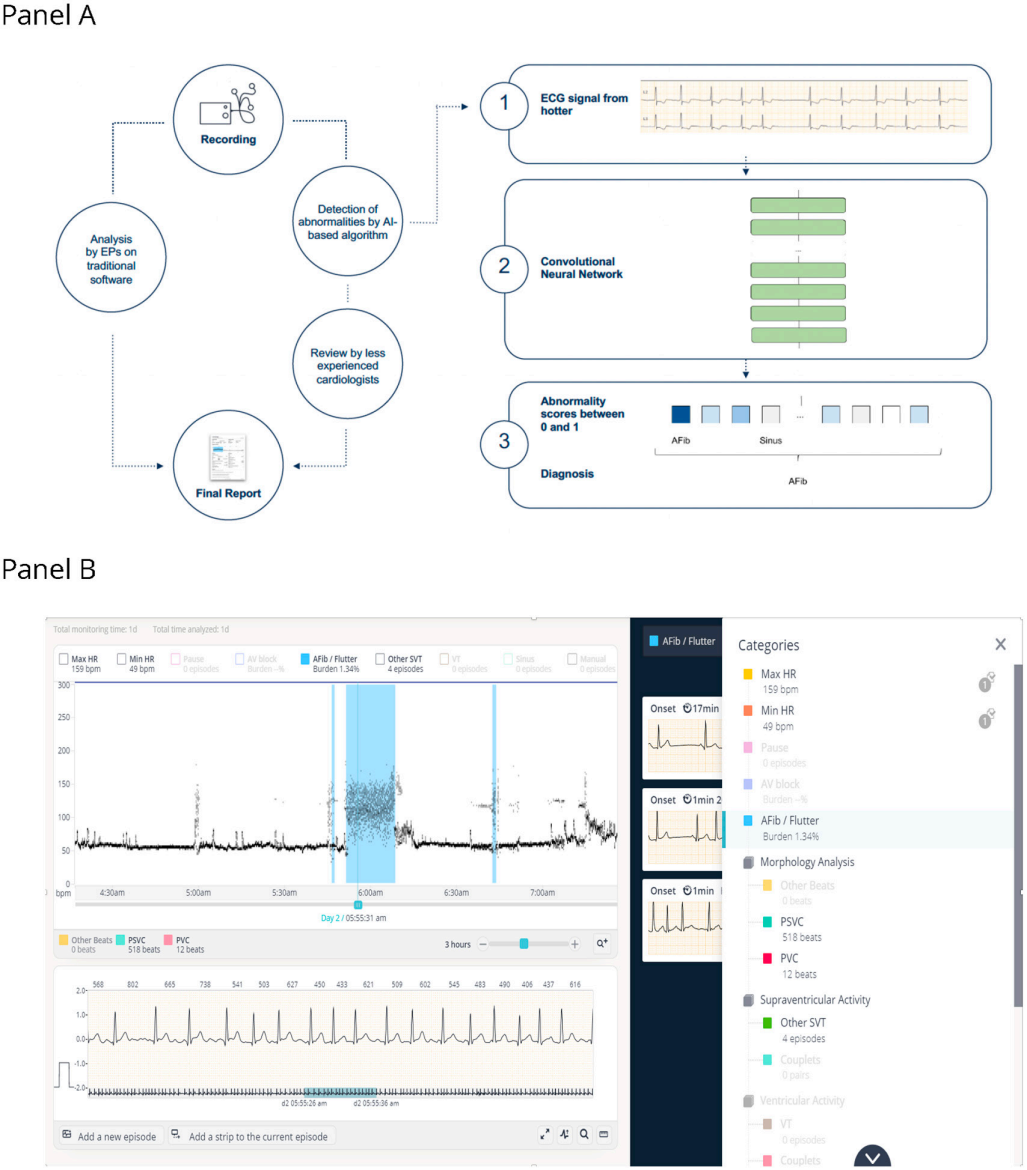
Study workflow and DNN platform overview. (A) Overview of the study workflow. (B) Screenshot of the DNN‐based platform where arrhythmia categories, heart rhythm plot, and full ECG are used by the physician to validate the analysis. AI indicates artificial intelligence; and EP indicates electrophysiologist.

The Cardiologs Holter analysis platform is a cloud‐based and DNN‐based platform (Figure 1A). Compared with a conventional ECG analysis platform, the cloud‐based access allows frequent automatic updates and access from any computer connected to the Internet, where multiple users can collaborate. This DNN‐based platform is also a vendor‐neutral solution, compatible with various recording formats, allowing use of a single software for various Holters or patches. As described in Figure 1, Holters are uploaded through a secured/encrypted connection, and ECGs are analyzed automatically. ECGs are first preprocessed using wavelet filtering to remove baseline wander and high‐frequency artifacts. Then, DNNs perform beat detection and rhythm classification. The physician can then review the Holter using an online platform (Figure 1B), with access to the signal, a representation of the heart rate over time, and a list of episodes proposed by the DNN interpretation that he or she can modify if needed.

### Deep Neural Network

The platform uses 2 different neural networks, one for wave detection and one dedicated to rhythm classification. The wave detector is a DNN with a U‐net architecture^7^ with 800 000 parameters, consisting of 11 convolutional layers and 6 residual blocks, similar to the wave detector described in a previous study.^8^ This network uses the ECG signal as input and outputs the onsets and offsets of P waves, QRS complexes, and T waves. The rhythm classifier is a DNN with a visual geometry group‐like architecture^9^ consisting of 4M parameters and 16 layers: 13 convolutional layers followed by 3 fully connected layers, similar to the DNN used in previous studies.^5^, ^10^ The rhythm predictor outputs the presence of multiple labels (sinus rhythm, atrial fibrillation [AF] or atrial flutter, other supraventricular tachycardia, atrioventricular block, ventricular tachycardia, noise) for every 10 seconds of the recording. Both neural networks were trained and validated using a data set of >1 million ECGs from a private anonymized data set, which had previously been adjudicated by physicians or certified ECG technicians. Training was achieved using stochastic gradient descent, early stopping, and dropout^11^ to avoid overfitting. The neural networks were implemented using the Keras framework with a Tensorflow back end (Google; Mountain View, CA) on K‐80 (Nvidia, Santa Clara, CA) graphics processing units.

### Holter Annotation

Holters were analyzed by experienced electrophysiologists with the conventional platform as part of normal clinical practice. The second analysis with the DNN‐based platform was performed by a second group of less experienced cardiologists (<5 years of experience after training), who were blinded to the results of the first analysis. Analysis time was recorded by each physician in both cases. The presence or absence of 5 prespecified arrhythmias was analyzed: pauses (≥2.5 seconds), AF, flutter, or tachycardia, combined into a same category^12^ (AF, ≥30 seconds), ventricular tachycardia (≥4) beats with at least 1 RR interval (<500 milliseconds), premature ventricular contractions with a burden ≥10%, and second‐degree Mobitz II or third‐degree atrioventricular block.

The ability to diagnose these 5 prespecified arrhythmias was assessed for each platform. The large number (1000) and relatively long duration (≥24 hours) of Holter recordings hindered expert electrophysiologists from reviewing the entire recording signal by hand, independently of any platform, in a timely manner. The reference diagnostic (ground truth) was therefore determined using the interpretation of both platforms. In case of discrepancy on any prespecified arrhythmia between both platforms, an expert electrophysiologist reviewed both analyses and adjudicated the rhythm. Because both platforms can miss abnormalities, only a combination of the two can be used to evaluate the quality of the analysis in a way fair to both platforms. While there is still a possibility for an abnormality to be missed by both platforms, thus resulting in an overestimation of the sensitivity of the platform, this method allows for a rigorous estimation of the differences of errors. In addition, 5% of randomly selected Holters without discrepancies were fully verified by one electrophysiologist who had the Holter analyses of both platforms available.

### Statistical Analysis

Quantitative variables were described by median (first, third quartiles), minimum and maximum because of the non‐Gaussian distributions. Categorical variables were described by number and percentage. The AI‐based method would be considered noninferior to the classical one for detection of major rhythm abnormalities if the lower bound of the one‐sided 97.5% CI of the difference between the detection rate of the 2 strategies was less than the noninferiority margin, which was set at 10%. Sensitivity, specificity, positive predictive value and negative predictive value for each major arrhythmia type with nonparametric 95% CI were also reported for both strategies and compared. The time needed for the analysis was compared between both platforms using a paired Student *t*‐test. All comparative tests were 2‐sided with a significance level of 5%. Analyses were performed with SAS software (SAS Institute Inc, Cary, NC).

## RESULTS

**Figure 2:**
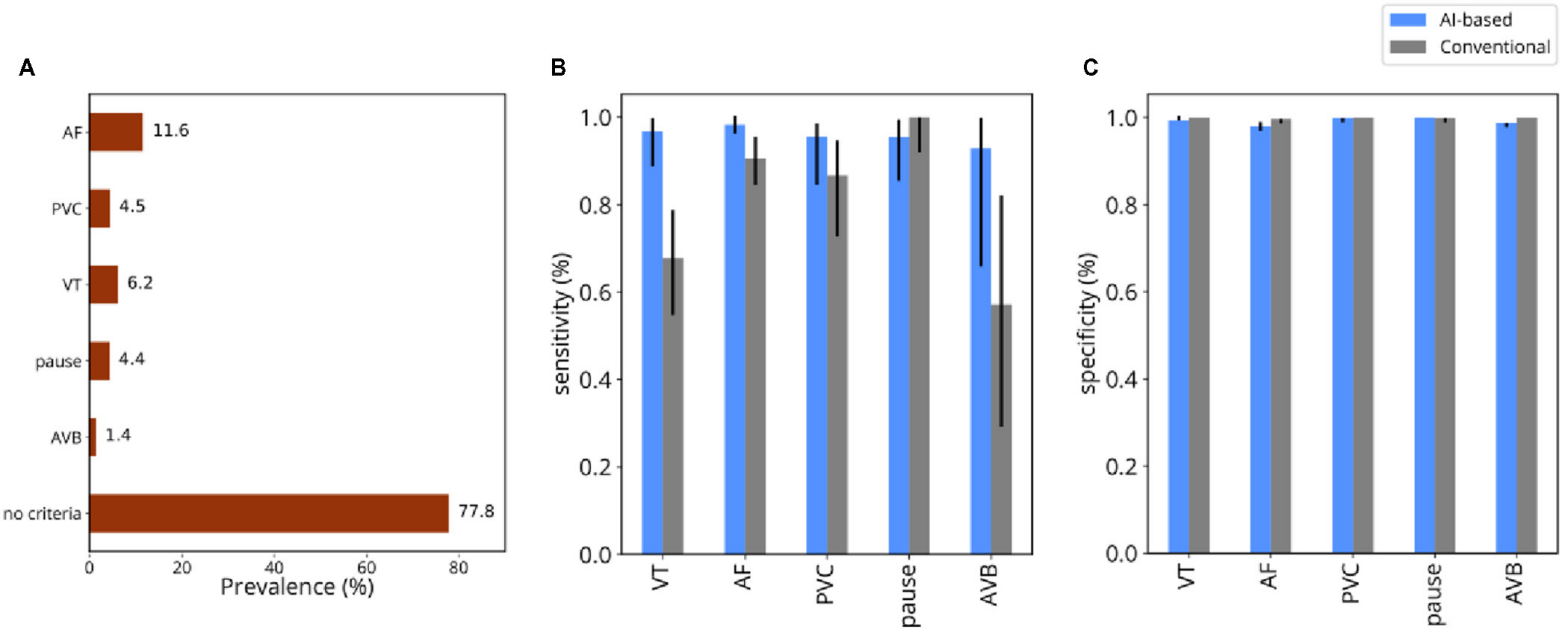
Arrhythmia distribution in the dataset and detection performance of the AI‐based and the conventional analysis platforms (A) Arrhythmia prevalence and (B) platform sensitivity and (C) specificity. Error bars correspond to 95% CI. AF indicates atrial fibrillation; AVB, atrioventricular block; PVC, >10% of premature ventricular contraction; and VT, ventricular tachycardia.

The defined major arrhythmias were present in 22.2% of the cases (Figure 2A).

The DNN‐based platform was noninferior to the conventional one in its ability to detect a major rhythm abnormality (95.6% of cases versus 95.6% difference CI, [−2.1% to 2.1%]). The sensitivities and specificities (Figure 2B and 2C) for the detection of each rhythm abnormality were not different for most rhythm disturbances. However, the DNN‐based platform had higher sensitivity for detecting AF (98% [96–100] versus 91% [85–96]; *P*=0.01) and ventricular tachycardia (97% [89–100] versus 68% [55–79]; *P*<0.001), while the classical analysis had slightly higher specificity for AF detection although of limited clinical relevance (100% [99–100] versus 98% [97–99]; *P*=0.001).

**Figure 3:**
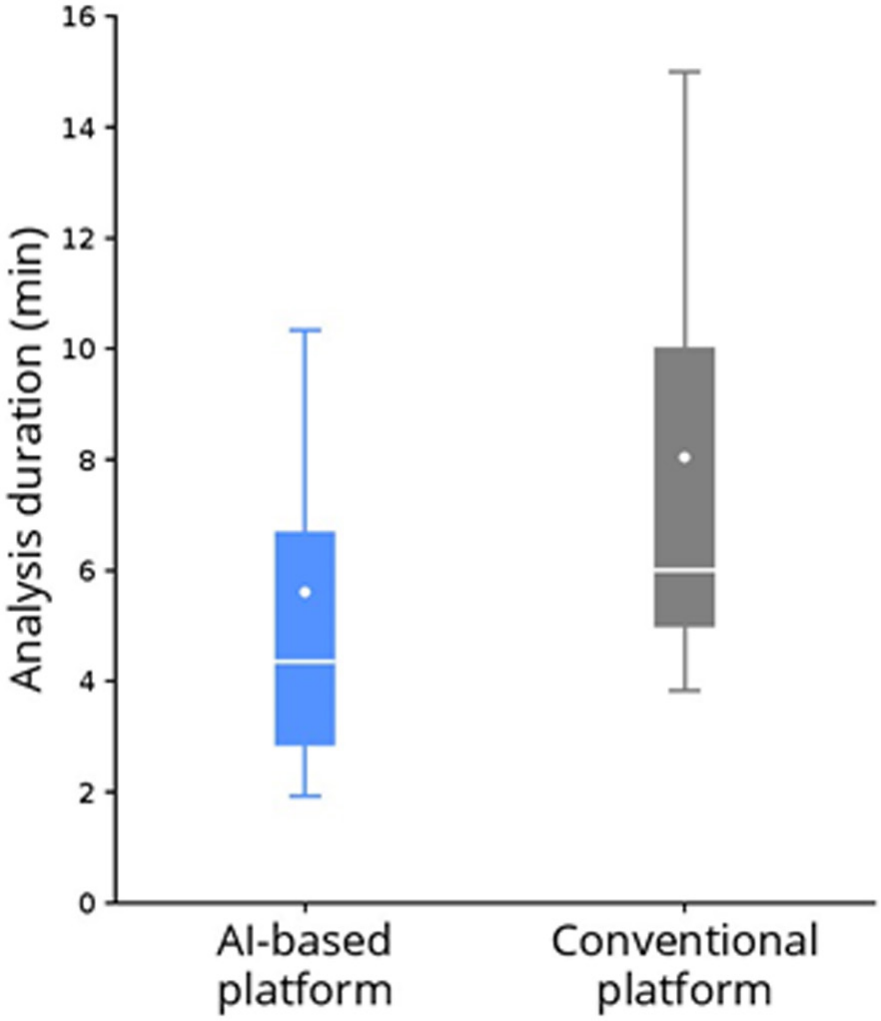
Analysis time. AI indicates artificial intelligence.

We compared the duration required to complete the analysis by the reading physicians, which was available for both platforms for 461 recordings. The median analysis time was significantly shorter with the DNN‐based platform compared with the standard platform with a 26.6% reduction: 4.4 minutes (interquartile range, 2.9–6.7 minutes) versus 6.0 minutes (5.0–10.0 minutes, *P*<0.001) (Figure 3).

## DISCUSSION

To the best of our knowledge, this is the largest study evaluating the performance of a DNN‐based platform for Holter analysis. We found that the DNN‐based platform was noninferior to the classical strategy in terms of overall diagnostic sensitivity and specificity, with the analysis duration being significantly shorter by 27% on average. The higher ventricular tachycardia and AF sensitivities of the DNN‐based platform were mostly attributable to premature ventricular contraction quadruplets and short episodes of AF being missed by the classical strategy. The lower specificity of DNN for AF was mostly attributable to rare episodes of sinus tachycardia being misclassified as AF by the DNN‐based platform. Importantly, there were no more significant false negatives compared with the classical strategy.

One of the key advantages with DNN is that its performance typically improves with increasing number of recordings analyzed; hence, accuracy is likely to improve in the future, with a subsequent reduction in time needed for manual overread. This is important, as the considerable time needed for analyzing Holters is still a concern and represents a significant burden on limited clinical staff, potentially delaying important results and limiting the wider adoption of this test, especially for stroke prevention.^13^

Previous studies have shown superiority of DNN over standard algorithms for 12‐lead ECG analysis in specific arrhythmias such as AF^5^ or in contexts such as the emergency department.^10^ In their study, Hannun et al^5^ showed impressive superiority of AI compared with a panel of cardiologists.

With regard to the actual use of AI in medical technologies, patients and physicians could be apprehensive about a partly automated tool that could make errors and the risk–benefit balance is an important consideration.^14^ Patients' confidence in AI use also depends on the extent of human control over it. Our study suggests that this new tool, with human oversight, performs well without increasing error rate. Progressively expanding use of AI and accumulating data will enhance patient and physician confidence for incorporating AI in clinical practice. Overall, DNN is a quickly evolving and promising technology that should enable improved outcomes for patient diagnosis and care in the near future. Improved algorithms should allow for even more accurate automated diagnosis and even lower overread time.

A limitation of our study is that all readers were specialized cardiologists and it is possible that results may differ with other readers (general cardiologist, physician, ECG technician) or longer recordings (reader fatigue). Another limitation was related to the experience of the physicians, which was different in the group using each platform. Cardiologists overreading the DNN‐based platform received only a 1‐day training on this platform and were less experienced than the group of electrophysiologists who performed the overreading of the conventional platform analysis, with which they were familiar. This might overestimate the standard platform performance and underestimate the analysis time reduction observed with the DNN‐based platform compared with prior studies.^15^ However, the fact that the DNN‐based platform performed at least as well as the standard approach despite less experienced cardiologists strengthens its validity. Finally, different results might be observed with a more recent version of the DNN‐based platform than the one used in the study (released in 2019).

In conclusion, our findings demonstrate that the use of a deep learning approach can help classify a broad range of distinct arrhythmias from ambulatory ECGs with high diagnostic performance. DNN‐based platforms hold promise to provide accurate, time‐efficient, and, ideally, fully automated Holter analysis in the future.

## Sources of Funding

The French National Institute of Health and Medical Research (INSERM).

## Disclosures

Dr Fiorina is a medical expert for Cardiologs. Dr Gardella, A. Plesse, and C. Henry are Cardiologs employees, and J. Li is a Cardiologs cofounder. Other authors have no disclosures to report.
